# Genomic and transcriptomic landscapes of metastatic neuroendocrine neoplasms from distinct primary sites and their clinical implications

**DOI:** 10.1038/s41598-025-00549-7

**Published:** 2025-05-06

**Authors:** Kathleen Wee, Kevin C. Yang, David F. Schaeffer, Chen Zhou, Emily Leung, Xiaolan Feng, Janessa Laskin, Marco A. Marra, Jonathan M. Loree, Sharon M. Gorski

**Affiliations:** 1 Canada’s Michael Smith Genome Sciences Centre, BC Cancer, Vancouver, BC Canada; 2 Department of Pathology and Laboratory Medicine, BC Cancer, Vancouver, BC Canada; 3 Division of Anatomic Pathology, Vancouver General Hospital, Vancouver, BC Canada; 4 Pancreas Centre BC, Vancouver, BC Canada; 5 Vancouver Island Centre, BC Cancer, Victoria, BC Canada; 6 Division of Medical Oncology, BC Cancer, Vancouver, BC Canada; 7 Department of Medical Genetics and Michael Smith Laboratories, University of British Columbia, Vancouver, BC Canada; 8 Department of Molecular Biology and Biochemistry, Simon Fraser University, Burnaby, BC Canada

**Keywords:** Neuroendocrine neoplasms, Genomics, Personalized medicine, Transcriptomics, Cancer genomics, Cancer, Computational biology and bioinformatics, Molecular medicine, Oncology

## Abstract

Neuroendocrine neoplasms (NENs) encompass a highly heterogeneous group of neoplasms with varying prognoses and molecular alterations. Molecular profiling studies have furthered our understanding of NENs, but the majority of previous studies have focused on primary tumors and on mutational landscapes using DNA sequencing data. Here, we describe the genomic and transcriptomic landscapes of 28 metastatic NENs across different primary anatomical sites (PASs) and their potential clinical implications. Although our cohort is small, our analyses provide further insights on the molecular commonalities and distinctions between metastatic NENs of different PASs. Comparison to several reference transcriptome data sets revealed that despite considerable whole genome and transcriptome variability in NENs, the metastatic NENs are still more like each other than other cancer types. Our study also highlights the potential utility of NEN transcriptome data for molecular classification and clinical decision making.

## Introduction

Neuroendocrine neoplasms (NENs) represent a diverse group of neoplasms arising from neuroendocrine cells that can be found in most tissues. Clinical features, such as histological differentiation and proliferative indices, are often used across primary anatomical sites (PASs) for prognostic and therapeutic purposes^1,2^ and generally dichotomize NENs into well-differentiated neuroendocrine tumours (NETs) or poorly-differentiated neuroendocrine carcinomas (NECs). NETs are further classified into grades 1 ~ 3 (G1 ~ 3) based on proliferative indices and NECs are further classified into small cell or large cell type^3^.

Although molecular studies have furthered our knowledge of NENs, most studies have focused on primary NENs of particular PASs. One study examined metastatic NENs across multiple PASs but was limited to utilizing genomic data^4^. Furthermore, molecular studies on mixed neuroendocrine non-neuroendocrine neoplasms (MiNENs) are extremely limited given their rarity. Transcriptomic comparisons may provide additional insights into the molecular characteristics of metastatic NENs of different PASs and potentially aid clinical management of this tumour group that currently has relatively limited standard treatment options.

We previously described the molecular architectures of six metastatic pancreatic neuroendocrine neoplasms (PanNENs)^5,6^, sequenced as part of the Personalized OncoGenomics (POG) program^7^ at BC Cancer. To investigate the potential similarities and differences between metastatic NENs across PASs and their clinical implications, we leveraged whole genome and transcriptome analysis (WGTA) from the POG program to describe the genomic and transcriptomic landscapes of a small cohort of NENs across PASs with metastatic disease and demonstrate their potential to impact clinical decision making.

## Results

**Fig. 1:**
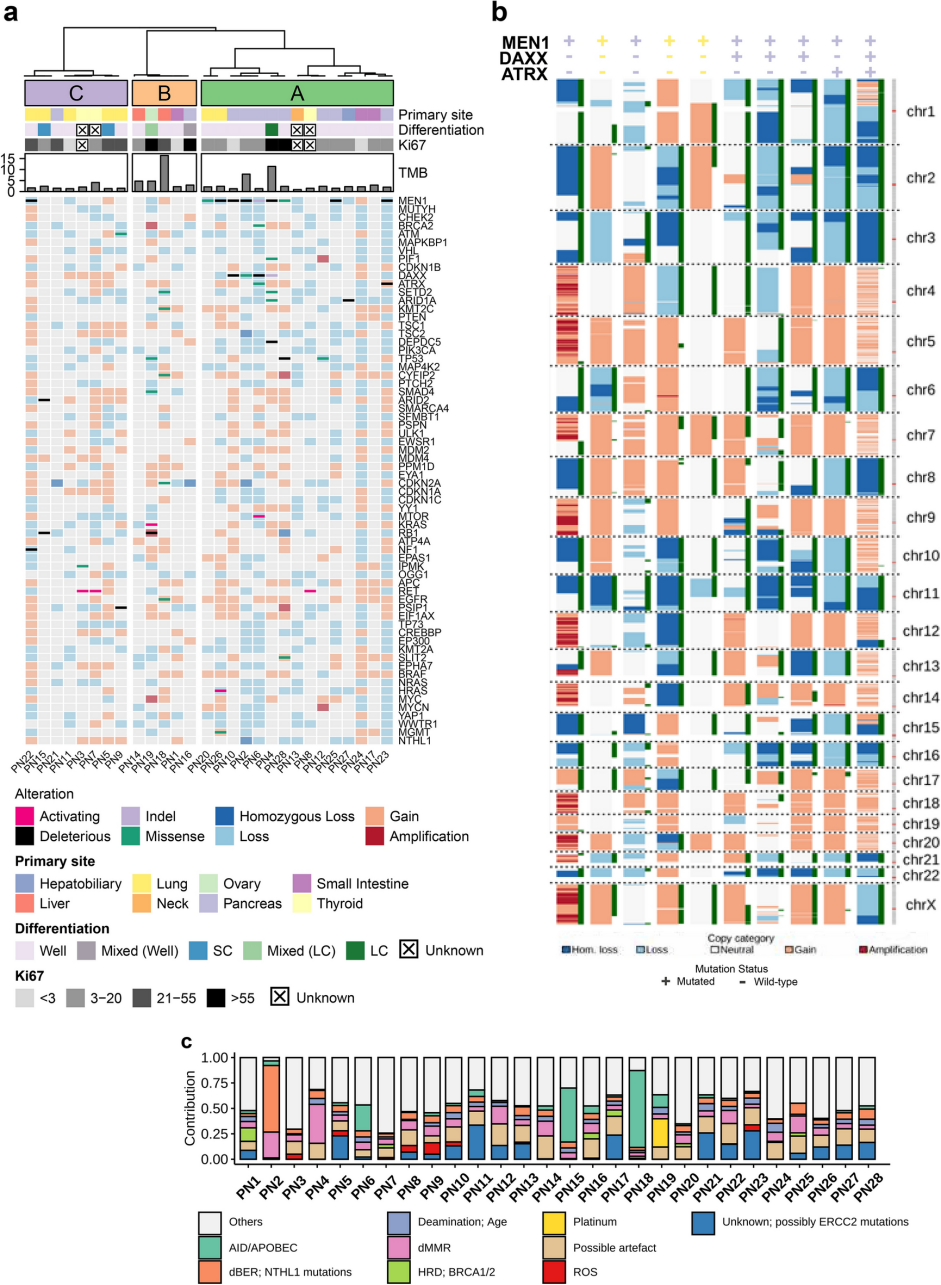
WGTA reveals molecular relationships between metastatic neuroendocrine neoplasms from distinct anatomical sites. (**a**) Overview of the clinicopathological and genomic characteristics of POG NENs. Samples are annotated based on cluster assignments, primary anatomical sites, histological differentiation, Ki-67 indices, and TMB. The oncoprint depicts the mutational and copy number status of NEN-associated genes manually curated from literature review. LC: large-cell; SC: small-cell. (**b**) Genome-wide copy number architectures of the 10 *MEN1*-mutant POG NENs reveal PAS-specific patterns of LOH. The copy number status (left; wide) and presence of LOH (right; slim) are depicted for each chromosome. The mutation status of *MEN1*, *DAXX* and *ATRX* are indicated at the top and colours reflecting the primary anatomical site of the case as in 1a. (**c**) Summary of mutational signature analysis against the COSMIC single base substitutions signatures. Signatures are combined based on their known associations and those that contributed to 10% or more of mutations in any sample are shown.

The cohort is comprised of 28 metastatic NEN patients who have undergone one or more treatments at the time of POG enrolment and included samples with eight different PASs and various pathological characteristics, a NEN of unknown primary and an ovarian MiNEN (Fig. 1a; Table 1; Supplementary Tables 1, 2).

**Table 1:** Summary of POG NEN primary anatomic sites and WGTA-informed treatments received by POG NEN patients.

StudyID	Primary anatomic Site	Actionable targets were identified	Received informed treatments, or why not	Treatments received	POG-informed agent	Treatment response	Treatment duration (days)
PN1	Small Intestine	Yes	Patient/physician choice				
PN2	Pancreas	Yes	Poor patient status				
PN3	Thyroid	Yes	POG informed	VANDETANIB	VANDETANIB	Stable disease	90
PN3	Thyroid	Yes	POG informed	LOXO-292	LOXO-292	Stable disease	1021
PN4	Pancreas	Yes	POG informed	CARBOPLATIN,IRINOTECAN	IRINOTECAN	Progressive disease	78
PN5	Lung	Yes	POG informed	OCTREOTIDE	OCTREOTIDE	Stable disease	117
PN6	Pancreas	Yes	POG informed	EVEROLIMUS	EVEROLIMUS	Stable disease	342
PN7	Thyroid	Yes	POG informed	SORAFENIB	SORAFENIB	Partial response	1098
PN7	Thyroid	Yes	POG informed	VANDETANIB	VANDETANIB	Progressive disease	64
PN7	Thyroid	Yes	POG informed	LOXO-292	LOXO-292	Stable disease	1423
PN8	Thyroid	Yes	POG informed	CABOZANTINIB	CABOZANTINIB	Partial response	164
PN9	Lung	No	Not actionable				
PN10	Pancreas	Yes	POG informed	SUNITINIB	SUNITINIB	Not evaluable	1
PN11	Lung	Yes	Patient/physician choice				
PN12	Pancreas	No	Not actionable				
PN13	Neck	Yes	Not available				
PN14	Unknown	Yes	POG informed	SUNITINIB	SUNITINIB	Stable disease	320
PN15	Lung	Yes	Unknown				
PN16	Pancreas	Yes	Deceased				
PN17	Small intestine	Yes	POG informed	EVEROLIMUS	EVEROLIMUS	Progressive disease	33
PN18	Hepatobiliary	Yes	POG informed	IRINOTECAN	IRINOTECAN	Progressive disease	70
PN19	Ovary	Yes	Deceased				
PN20	Lung	No	Not actionable				
PN21	Pancreas	Yes	POG informed	EVEROLIMUS	EVEROLIMUS	Progressive disease	25
PN22	Lung	Yes	Not available				
PN23	Pancreas	Yes	POG informed	EVEROLIMUS	EVEROLIMUS	Stable disease	147
PN23	Pancreas	Yes	POG informed	LANREOTIDE,OCTREOTIDE	LANREOTIDE,OCTREOTIDE	Stable disease	1972
PN24	Small intestine	Yes	POG informed	SUNITINIB	SUNITINIB	Stable disease	207
PN25	Pancreas	Yes	Unknown				
PN26	Lung	Yes	POG informed	EVEROLIMUS	EVEROLIMUS	Stable disease	179
PN26	Lung	Yes	POG informed	LENVATINIB	LENVATINIB	Stable disease	98
PN26	Lung	Yes	POG informed	CAPECITABINE, TEMOZOLOMIDE	TEMOZOLOMIDE	Not evaluable	1
PN27	Hepatobiliary	Yes	Unknown				
PN28	Pancreas	Yes	POG informed	CAPECITABINE, TEMOZOLOMIDE	TEMOZOLOMIDE	Partial response	270
PN28	Pancreas	Yes	POG informed	LANREOTIDE	LANREOTIDE	Unknown	34

A manually curated list of recurrent alterations observed in NENs from various PASs was generated from the literature^5,6,8–17^ and used to identify relevant alterations in our metastatic NEN cohort. Recurrent deleterious *MEN1, DAXX, RB1,* and *TP53* mutations were observed in pancreatic (PanNENs) and pulmonary (PulNENs) neuroendocrine neoplasms while activating *RET* mutations were found in medullary thyroid carcinomas (MTCs) (Fig. 1a; Supplementary Table 3). The majority of the *MEN1*-mutant PanNENs were affected by large scale loss of heterozygosity (LOH) while *MEN1*-mutant PulNENs were not (Fig. 1b). The median tumour mutation burden (TMB) in our metastatic cohort was 2.19 mutations per megabase (mut/mb), with two cases having a TMB greater than ten (range: 0.89 ~ 16.40; Fig. 1a; Supplementary Table 1). Aneuploidy was common regardless of the PAS, and recurrent copy number alterations were observed in several cases. Copy gains were common in certain chromosomes; however, amplification events were infrequent, with only PanNET PN28 observed to have large-scale amplification throughout multiple chromosomes (Supplementary Fig. 1a). Amplification of *MYC* or *MYCN* was observed in an ovarian and pancreatic NEN, the latter of which we previously characterized^5^.

Analysis of COSMIC mutational signatures^18^ revealed substantial contributions from the APOBEC family of cysteine deaminases (AID/APOBEC) and DNA mismatch repair deficiency (dMMR and DNA replication slippage) in multiple cases (Fig. 1c; Supplementary Table 4), with PN12 demonstrating evidence of potential kataegis (Supplementary Fig. 1b). Of note, two samples exhibited a considerable number of mutations likely driven by a DNA repair-related genetic alteration or prior treatment: PN2 was previously described with germline biallelic loss of *NTHL1*^5^ and had a strong dMMR signature, while PN19, who received platinum therapy for approximately 11 months prior to biopsy, exhibited mutational signatures associated with prior platinum therapies (Supplementary Table 2). While multiple dMMR-related mutation signatures were observed at low frequency across the cohort, dMMR signatures contributed to more than 20% of mutations only in PN2 and PN4. Mutations in mismatch repair genes were only observed in PN1 and PN4 and none were predicted to be microsatellite instability (MSI)-high. Homozygous loss of function mutations were observed in *MSH6* and *MLH1* in PN4 and 18% of microsatellites were unstable but were predicted to be MSI-low while a heterozygous variant of unknown significance in *MSH3* was observed in PN1 and was predicted to be MSI-stable.

**Fig. 2:**
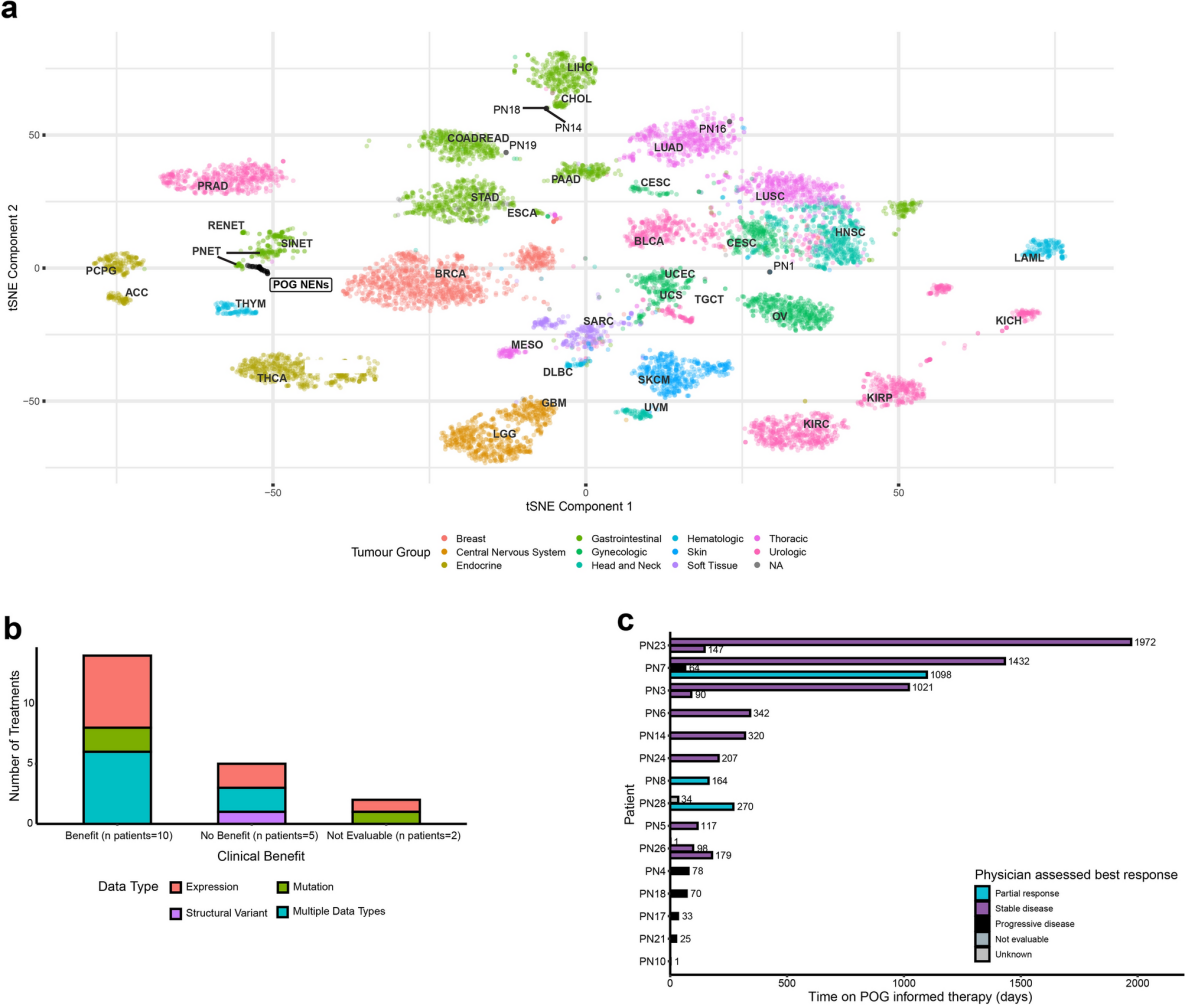
Whole transcriptome analysis reveals relationships of advanced metastatic NENs to primary tumours and enhances identification of potential therapeutic options in the clinic. (**a**) t-SNE results for the combined analysis of POG NENs, TCGA tumours, and tumours from public NEN cohorts. Each dot represents a tumour sample and is colour-coded based on its tumour group while POG NENs are depicted in black. (**b**) Treatment outcomes based on data type (Benefit = stable disease, partial response; No Benefit = progressive disease). “Multiple” indicates at least two data types were used in combination to help inform treatment. (**c**) Time on treatment for patients who received POG informed therapies and best response achieved as assessed by the treating oncologist.

Unsupervised clustering of POG NEN transcriptome profiles robustly partitioned the cohort into three groups (Fig. 1a; Supplementary Fig. 2). Cluster A contained the majority of the small intestinal NETs and PanNETs, where we observed the majority of *MEN1*-mutant PanNEN cases and all cases with *DAXX* and/or *ATRX* mutations. Cluster C consisted primarily of PulNETs and MTCs. NENs of various PASs comprised Cluster B and were generally characterized as high grade (Ki67 > 20%) and/or with poorly-differentiated or mixed histology. Interestingly, seven NENs belonging to Clusters A or C were NECs or NET-G3s. This observation is consistent with principal component analysis results demonstrating that NECs did not form a distinct cluster from NETs in our cohort (Supplementary Fig. 3a). Clustering analysis using a set of 1,553 genes^7^ that was previously identified to best discriminate between tumour types revealed that 82% (23/28) of the POG NENs formed a distinct group in close proximity to NENs from external datasets^8,15^ and were distinct from other types of primary tumours from The Cancer Genome Atlas (TCGA) (Fig. 2a). However, when comparing the transcriptome profiles of metastatic POG NENs with gastroenteropancreatic (GEP)^15^ and pancreatic^8^ NEN datasets, all POG cases clustered together (Supplementary Fig. 3b).

Intriguingly, POG NENs belonging to Cluster B clustered based on their PAS (PN14 and PN18) or other non-related tumour types (PN1, PN16 and PN19). For example, ovarian MiNEN PN19 is a high-grade sample with a *MYC* amplification that clustered with TCGA colorectal adenocarcinomas (Fig. 2a). This observation was consistent when spearman correlation analysis was used to characterize and compare its transcriptome profile to TCGA reference data sets (Supplementary Fig. 3c). Gene set enrichment analysis on Cluster B samples identified enrichment of MYC targets (Supplementary Table 5) gene sets supporting the effects of the observed *MYC* amplification. Master regulator analysis also confirmed the relative activation of MYC family genes in Cluster B, while *MEN1* and *DAXX* were relatively inhibited in Cluster A (Supplementary Table 6), consistent with the recurrent loss of *MEN1* and *DAXX* in this cluster.

As part of the POG program, WGTA is utilized to gain insight into metastatic/advanced disease and may also provide insight into diagnosis and treatment options on a personalized level. In this cohort, WGTA resulted in the identification of alterations considered potentially clinically actionable in 24 patients and consisted of small nucleotide, copy number, structural variants, expression outliers, and genomic signatures (Table 2). Of these, 15 patients went on to receive standard of care treatments that were supported by WGTA findings, 10 of whom experienced clinical benefit as defined by the treating clinician’s assessment of treatment response, which was also associated with longer time on treatment (Fig. 2b,c; Table 1). The data types which informed the majority of the treatment options received were based on expression findings, mutation findings or both (Fig. 2b; Table 2).

**Table 2:** Summary of all somatic alterations identified as clinically actionable in the POG NEN cohort.

Action #	Study ID	Clinical action	Action rationale				
			Genome signature	Small mutation	Copy number variant	Structural variant	RNA expression
1	PN1	Sorafenib					↑RET,PIK3R2
2	PN1	Pazopanib			FGFR3 gain		↑RET,FGFR3,PIK3R2
3	PN1	HDAC inhibitor			HDAC1,HDAC3,HDAC5,HDAC9, HDAC10 gains		↑HDAC1,HDAC3,HDAC5,HDAC9,HDAC10
1	PN2	Sunitinib					↑FLT1,KDR,PDGFRA,PDGFRB
2	PN2	Temozolomide					↑MGMT
1	PN3	RET inhibitor		RET GoF			↑RET
1	PN4	Irinotecan			TOP2A amplification		↑TOP1,TOP2A
2	PN4	Everolimus					↑RICTOR
3	PN4	Sunitinib					↑RET
1	PN5	Immune checkpoint inhibitor					↑CD274
2	PN5	Somatostatin analog					↑SSTR1
1	PN6	Everolimus		MTOR GoF			
1	PN7	Sorafenib		RET GoF			↑RET
2	PN7	Somatostatin analog					↑SSTR1,SSTR2,SSTR3,SSTR4,SSTR5
1	PN8	Cabozantinib		RET GoF			↑RET,HGFAC
2	PN8	Vandetanib		RET GoF			↑RET,HGFAC
1	PN10	Sunitinib					↑PDGFRB,FLT1,KDR,KIT
2	PN10	Somatostatin analog					↑PDGFRB,FLT1,KDR,KIT
1	PN11	Irinotecan					↑TOP1
2	PN11	DLL3 inhibitor					↑DLL3
1	PN13	Tyrosine kinase inhibitor					↑FGR4,FGFR3,EGFR,MET,JAK3,JAK1,PDGFA,PDGFB,PDGFC, PDGFRA,PDGFRB,KRAS,NRAS,BRAF,MYB,MYCN,PIK3CA
2	PN13	MET inhibitor					↑MET
1	PN14	Sunitinib					↑FLT1,KDR,FLT4
2	PN14	FGFR3 inhibitor					↑FGFR2,FGFR3
1	PN15	MET inhibitor					↑MET
1	PN16	CDK4/6 inhibitor			CDKN2A,CDKN2B HOMD		
1	PN17	FGFR3 inhibitor					↑FGFR3
2	PN17	Sunitinib or Everolimus					↑PIK3C2G,MTCP1
1	PN18	Immune checkpoint inhibitor	↑TMB				↑CD274
2	PN18	Irinotecan-based therapy					↑TOP1,CES2
1	PN19	Immune checkpoint inhibitor	SBS4				↑CD274,PDCD1
2	PN19	DLL3 inhibitor					↑DLL3
1	PN21	MTOR inhibitor				TSC1-TMEM71 fusion	
1	PN22	DLL3 inhibitor					↑DLL3
2	PN22	Cell cycle inhibitor		MEN1 LoF			↓CCND1,CDKN1A
1	PN23	MTOR inhibitor		MEN1 LoF			↓MEN1
2	PN23	Somatostatin analog					↑SSTR1, SSTR2
1	PN25	Tyrosine kinase inhibitor					↑EGFR,FGFR3,KIT,KDR,MET,HGF,ERBB4
2	PN25	MTOR inhibitor					↑RICTOR
1	PN26	Lenvatinib					↑RET
2	PN26	MTOR inhibitor		MEN1 LoF			
3	PN26	Temozolomide		MGMT VUS			
1	PN27	PARP inhibitor		BRIP1 LoF			
1	PN28	Somatostatin analog					↑SSTR1,SSTR2,SSTR3,SSTR5
2	PN28	Temozolomide					↓MGMT

*GoF* gain of function mutation, *LoF* loss of function mutation, *HOMD* homozygous loss, *TMB* tumour mutation burden, =  ↑ high expression, ↓ low expression, *VUS* variant of unknown significance.

## Discussion

Because of their inherent heterogeneity, diagnosis and treatment of NENs remain significant clinical challenges, particularly in the metastatic setting. The POG NENs described in our work, despite the small cohort size, are clinically relevant as these are representative of what is observed in our tertiary care centre at BC Cancer. Molecular characterization through WGTA of these tumours further our understanding of metastatic NENs and potentially identify novel therapeutic approaches which remain a critical area of need in this tumour type.

Many of the genomic findings in our cohort are consistent with previous studies in primary NENs. The recurrent alterations observed in tumour suppressors known to be involved in tumourigenesis of PanNENs^9,10,15^ and PulNENs^8,11,12^, and activating mutations in oncogenes in MTCs^13,19^ further support their role as driver mutations in this tumour type. Although *MEN1* alterations were observed in both PanNENs and PulNENs in our cohort, LOH was only observed in *MEN1* altered PanNENs which is also consistent with the literature^20–23^. The median TMB of 2.19 mut/Mb that was observed in our metastatic cohort is also in line with previous findings in metastatic NENs, where a median TMB of 1.09 ~ 2.95 and 5.45 mut/mb have been reported in metastatic NETs and NECs, respectively^4,5,24^.

Mutational signature analysis revealed significant contributions from the APOBEC family of cysteine deaminases and deficiencies in DNA repair in POG NENs, including a germline *NTHL1* loss of function mutation in PN2 that was previously characterized by our group^5^. Similar to previous findings in metastatic NENs^4^, we also observed several POG NENs with dMMR signatures present; however, deleterious mutations in mismatch repair genes were only observed in PanNET PN4. While genome TMB was elevated at 11 mut/Mb, only 18% of microsatellites were unstable and resulted in an MSI-low prediction by MSIsensor. This is consistent with the literature, where MSI-high status has been observed in NETs of colonic origin but is uncommon in NENs of other PASs^25,26^. Furthermore, mismatch repair deficiency is not always synonymous with MSI-high. Jaffrelot et al. demonstrate that 15% of dMMR tumours can present with an unusual phenotype where they are not considered to be MSI-high, particularly in non-colorectal cancer tumours^27^. In some cases, loss of multiple mismatch repair genes still resulted in MSI-stable or MSI-low status.

To our knowledge, our work represents the first comparative investigation of metastatic NEN transcriptomes of various PASs. Comprehensive transcriptome analysis using various clustering methods and tools revealed that although metastatic NENs clustered largely, though not fully, based on PAS and histological differentiation, they cluster closely with primary pancreatic^9^ and both primary and metastatic GEP^28^ NEN samples compared to other tumour types. This result further suggests that NENs share biological characteristics at the transcriptome level and are distinct from other tumour types irrespective of PAS. Our tSNE analysis focusing on comparing POG NENs with GEP-NETs from Alvarez et al.^28^ also demonstrate that metastatic NENs can have different transcriptome profiles. Notably, POG NENs all had at least one prior line of systemic therapy while the GEP-NET samples were collected as surgical resections or as biopsies with sparse clinical history. Despite both cohorts having metastatic NENs with shared PASs, POG NENs still clustered together and away from the GEP-NET reference data set and we hypothesize that the systemic therapies received prior to sequencing may have impacted the transcriptomes of the POG NENs.

Molecular characterization of NET-G3s remain sparse^29^. Our observations of several NECs and NET-G3s that clustered with low grade NETs in clusters A and C suggest similarities at the transcriptome level between these poorly-differentiated/high grade cases and low grade NENs in a metastatic setting. However, with the exception of PN14 and PN18, the high-grade/poorly differentiated NENs that belong to Cluster B appear to have transcriptomes which are distinct from their PASs which we hypothesize may be a result of their poorly differentiated histology and also result in poorer patient prognosis. Based on pathway and gene set enrichment analysis, involvement of MYC targets suggests that Cluster B NENs may have more aggressive disease. Altogether, our transcriptome data suggest that metastatic NENs have a distinct molecular profile as a tumour group but appear to retain characteristics derived from their respective PASs and histological subtypes.

Availability of WGTA allows a wholistic investigation of very rare tumours as demonstrated by molecular characterization of ovarian MiNEN PN19. Ovarian MiNENs are extremely rare, and to our knowledge, comprehensive molecular characterization of these tumors remains sparse. The main genomic findings of this case included a *KRAS* gain of function mutation, loss of function mutations in *TP53*, *RB1*, *SMAD4*, and amplification of several oncogenes, namely *MYC*, *CDK8*, and *FLT1*. Notably, *MYC*-driven NENs are highly aggressive and resistant to treatments^14,30,31^ and may contribute to inferior survival outcomes. In line with these observations, PN19 had treatment refractory disease and passed away shortly following biopsy of the metastatic lesion. Interestingly, PN19 clustered with TCGA colorectal cancer cases, which we hypothesize may reflect the dominant histological subtype in this MiNEN, which is comprised of 60% adenocarcinoma and 40% neuroendocrine, as MiNENs may have histology components from multiple PASs^32^. The PN19 clustering pattern may also reflect shared genomic marks that may be due to similar treatment modalities^7^ in these tumour types as demonstrated by the presence of mutation signatures associated with prior platinum treatment.

In addition to investigating molecular characteristics of rare tumour types, WGTA has potential clinical utility by providing a personalized approach to identifying therapies. WGTA may result in both identification of genomic alterations which may lead to clinical trial eligibility and may also inform how to sequence standard therapies that are tailored to each patient. In our POG NEN cohort, including expression data into routine analysis resulted in six patients who received standard treatments that were informed by transcriptome data alone and experienced clinical benefit, which was defined clinically by the treating physician. These results are consistent with recent work suggesting that expression-based findings are equally informative in identifying potential therapeutic targets^33^.

In summary, we characterized the genome and transcriptome landscapes of 28 metastatic NENs across various PASs. In addition to our small cohort size, other limitations of our study include the inability to compare against pre-treatment samples due to the eligibility criteria for POG enrolment which emphasizes advanced/metastatic cancers^7^. Despite these limitations, we identified genomic alterations that were largely consistent with previous reports of primary and metastatic NENs. Inclusion of transcriptome data enabled us to gain molecular insights on shared and distinct biological characteristics of these rare tumours across PASs, particularly cases with high-grade/poorly differentiated histology and highlight their molecular complexity. Despite considerable WGTA variability in metastatic NENs from the same PAS, we found that the majority of NENs are still more similar to each other than to other cancer types. Although larger cohort studies are required, we highlight the potential that WGTA has in the clinic where it may help clinicians choose how to sequence therapies, which remains a major clinical challenge for NENs.

## Methods

### Identification and inclusion of metastatic NEN cases for molecular analyses

Over 1200 cases were referred to the personalized oncogenomics (POG) program^7^ between 2014 and April 2021. After selection for cases with 1) available WGS and WTS data, 2) a sequencing-estimated tumour content of 30% or more and 3) a NEN diagnosis or pathological indication of neuroendocrine components in the examined specimen, 28 cases were identified and included in the present study. All 28 cases were metastatic and the majority had undergone one or more treatments at the time of program enrolment. 60% of the patients included were male, and the median age of the patients was 53 (range: 24–75). The majority (26/28) of the tumour specimens obtained for WGTA were biopsied from metastases, and liver was the biopsy site in 71% (20/28) of the cases, including for one hepatic NEN (HepNEN) case. Biopsied specimens obtained from the patients supported the pathology of PanNENs in 10, six of which were previously described in detail^5,6^, PulNENs in seven, MTCs in three, and NENs of other PASs in the remaining 8 patients. The histopathological characteristics of the specimens were heterogeneous and included NETs, NECs of varying grades and a MiNEN of ovarian origin.

### Sample collection and processing

Following informed consent, patient samples were collected and processed as previously described^7^. Briefly, biopsies were obtained and embedded in optimal cutting temperature (OCT) compound. Tumour sections most suitable for DNA and RNA extraction were selected for sequencing analysis. Peripheral blood samples drawn from the patients were subjected to WGS and served as germline reference for identification of somatic molecular alterations.

### Nucleic acid extraction and library construction

DNA and RNA were extracted for library construction as previously described in detail^7^.

### Sequencing and bioinformatics

Paired-end reads were generated on the Illumina platform and aligned to the human reference genome (hg19) by the BWA aligner^34^ (v.0.5.7 and v.0.7.6a). Somatic small nucleotide variants (SNVs) and small insertions/deletions were detected using Strelka^35^ (v.1.0.6). Somatic copy number alterations were identified using CNASeq^36^ (v.0.0.6) and loss of heterozygosity by APOLLOH^37^ (v.0.1.1 and v.0.2.1). Structural variants were detected using de novo assembly of tumour reads using ABySS^38^ (v.1.3.4) and Trans-ABySS^38,39^ (v.1.4.10). Mutations were annotated to Ensembl v69^40^ using SNPEff^41^ (v.3.2). Tumour content, sequence length of paired end reads, and sequencing coverage for each case is outlined in (Supplementary Table 7).

### Gene expression analysis

RNA-seq reads were aligned using STAR^42^ (v.2.5.2b) to the human reference (hg38) (http://hgdownload.cse.uscs.edu/goldenPath/hg38/bigZips/) with gene annotations based on Ensembl v100^43^ and normalized expression levels were calculated in transcripts per million mapped reads (TPM) using RSEM^44^ (v.1.3.0). Publicly available transcriptome sequencing data from the TCGA (https://tcga-data.nci.nih.gov/tcga/) and two published gastroenteropancreatic NEN studies^9,28^ were used to investigate the biological characteristics of the POG NEN cohort using clustering and differential gene expression analyses.

### Transcriptome-based clustering

Unsupervised cluster analysis was performed using consensus hierarchical clustering to obtain robust clustering solutions that group the cases based on transcriptomic profiles. TPM values from protein-coding genes were log2-transformed and filtered to remove mRNAs expressed below 1 TPM in > 50% of the cohort. The log2-TPMs from the top 25% mRNAs, ranked based on median absolute deviation, were then converted into z-scores for cluster analysis. Consensus hierarchical clustering was performed using the R package ConsensusClusterPlus^45^ (v.1.50) using Spearman’s correlation for distance calculation with 1000 iterations for up to a max k of 7, 80% gene resampling, and 80% sample resampling. The cluster solution at the rank of 3 was chosen because it was the lowest rank at which the consensus and cluster confidence reached an approximate maximum (Supplemental Fig. 3).

The t-distributed stochastic neighbor embedding (t-SNE) decomposition plots were generated using default parameters as previously described^7^. Log2TPM values were used as input and an aggregated set of 1,553 genes were identified that best discriminated between all tumour types by pairwise-ANOVA and used as input to the decomposition.

### Mutational signature analysis and microsatellite instability

Somatic mutation signature analysis was performed as described previously^18^. Signatures were compared against their respective COSMIC reference signature (v.3, May 2019, https://cancer.sanger.ac.uk/cosmic/signatures/). MSI scores were determined using MSIsensor^46^ (v.0.2) as described previously^7^. Briefly, scores were computed from genome alignments as a percentage of total sites displaying MSI.

### Differential expression analysis

Differential gene expression analysis was performed using linear models as previously described^5^ with lowly expressed RNAs excluded using the *filterByExpr()* function from edgeR^47^ (v.3.28.1). Differential analysis was performed to compare between each cluster to the combination of the other two clusters. Gene set tests were performed using the resultant differential statistics against gene sets from the Molecular Signature Database^48^.

### Master regulator analysis

Activities of master regulators were inferred using VIPER^49^ with previously published NEN-specific regulon^50^. The statistics from differential analysis were used to construct the gene expression signature for each cluster thereby providing relative activities of master regulators in each cluster.

## Supplementary Information

Supplementary Information 1.

Supplementary Information 2.

Supplementary Information 3.

Supplementary Information 4.

Supplementary Information 5.

Supplementary Information 6.

Supplementary Information 7.

Supplementary Information 8.

Supplementary Information 9.

## Data Availability

DNA and RNA sequencing data have been deposited in the European Genome-phenome Archive (EGA) as part of the study EGAS00001001159 with accession numbers as listed in (Supplementary Table 8).
