# Supplementary material for: Genomic and transcriptomic landscapes of metastatic neuroendocrine neoplasms from distinct primary sites and their clinical implications
Source: Sci Rep. 2025 May 6;15:15770. doi: 10.1038/s41598-025-00549-7 (PMC12056210; doi:10.1038/s41598-025-00549-7)

Genomic and transcriptomic landscapes of metastatic neuroendocrine neoplasms from distinct primary sites and their clinical implications

Kathleen Wee, Kevin C Yang, David F Schaeffer, Chen Zhou, Emily Leung, Xiaolan Feng, Janessa Laskin, Marco A Marra, Jonathan M Loree, Sharon M Gorski

Supplementary Figure 1. Copy number alterations and large structural rearrangements observed in the 28 POG NENs.

a. Genome-wide copy number architectures for the 28 POG NENs demonstrates recurrent loss of heterozygosity in metastatic NENs. For each case (rows), the copy number status (top; wide) and presence of loss of heterozygosity (bottom; slim) are depicted for chromosomes 1-22 and X (left to right).
b. Circos plot demonstrating large structural rearrangements in PN12 largely involving chromosomes 2 and 15.

Supplementary Figure 2. Unsupervised cluster analysis using consensus hierarchical clustering identifies 3 clusters from the 28 POG NENs.

a. The consensus cumulative distribution function for each k tested.
b. Delta area plot demonstrating the relative change in area under cumulative distribution function at each increment of k.
c. The consensus heatmap at k = 3; the intensity of the blue represents increasing consensus.
d. The consensus heatmap at k = 4.
e. The consensus heatmap at k = 5.
f. A tanglegram comparing cluster assignments between k = 3 and k = 5 showing that one cluster from k = 3 is subdivided into 3 clusters at k = 5.

Supplementary Figure 3. Whole transcriptome analysis reveals relationships of advanced metastatic NETs with NECs and other published GEP-NET reference data sets.

a. Principal component analysis results for metastatic POG NENs.
b. Analysis of POG NENs with external NEN cohorts using t-distributed stochastic neighbor embedding shows that POG NENs cluster closely with external NENs but largely form a distinct cluster. Metastatic POG NENs were compared to primary and metastatic gastroenteropancreatic NENs from Alvarez et al. (2018).
c. Spearman correlation results for PN19 demonstrating strong correlation with TCGA colorectal samples (COADREAD) at the transcriptome level. The red vertical line corresponds to the transcriptome profile for PN19 and the orange bars represent the correlation results with the reference TCGA tumour types while the blue bars represent the correlation results with reference normal tissue. The number of samples for each TCGA tumour type ("cancer") and normal tissue ("normal") reference data set is noted on the right.

Supplementary Figure 1

a

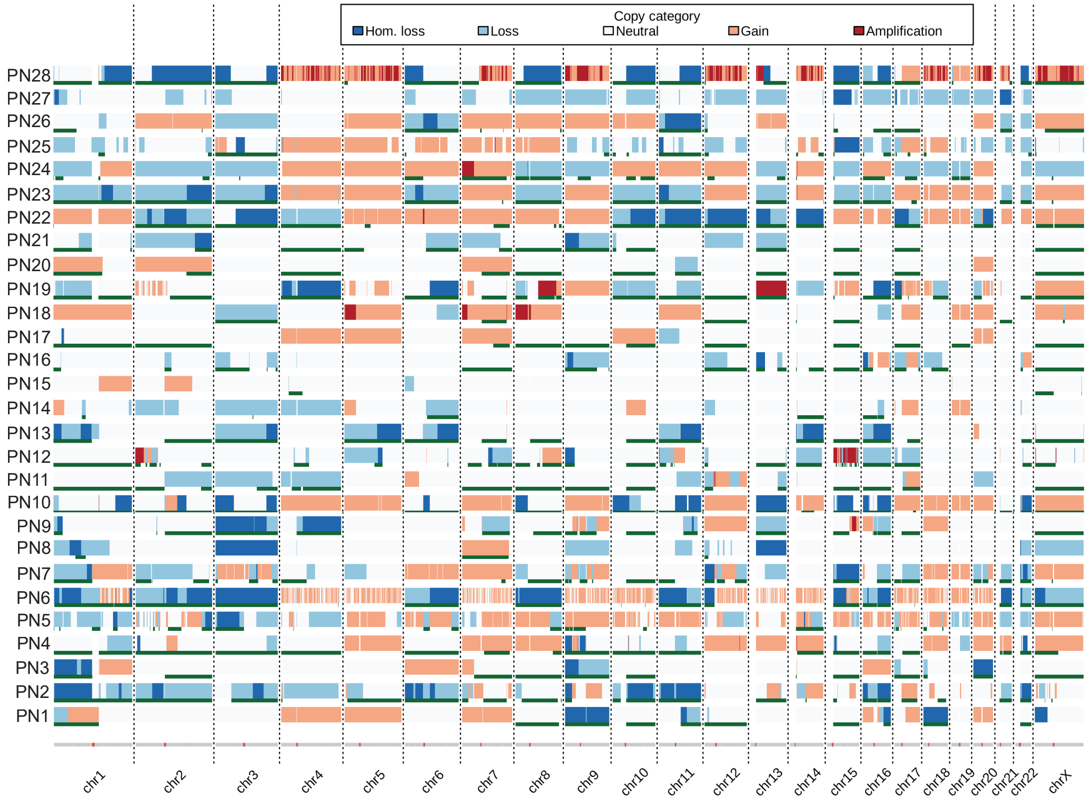

b

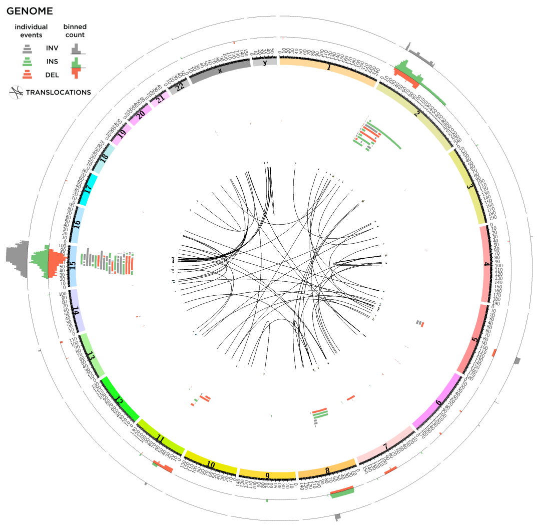

Supplementary Figure 2

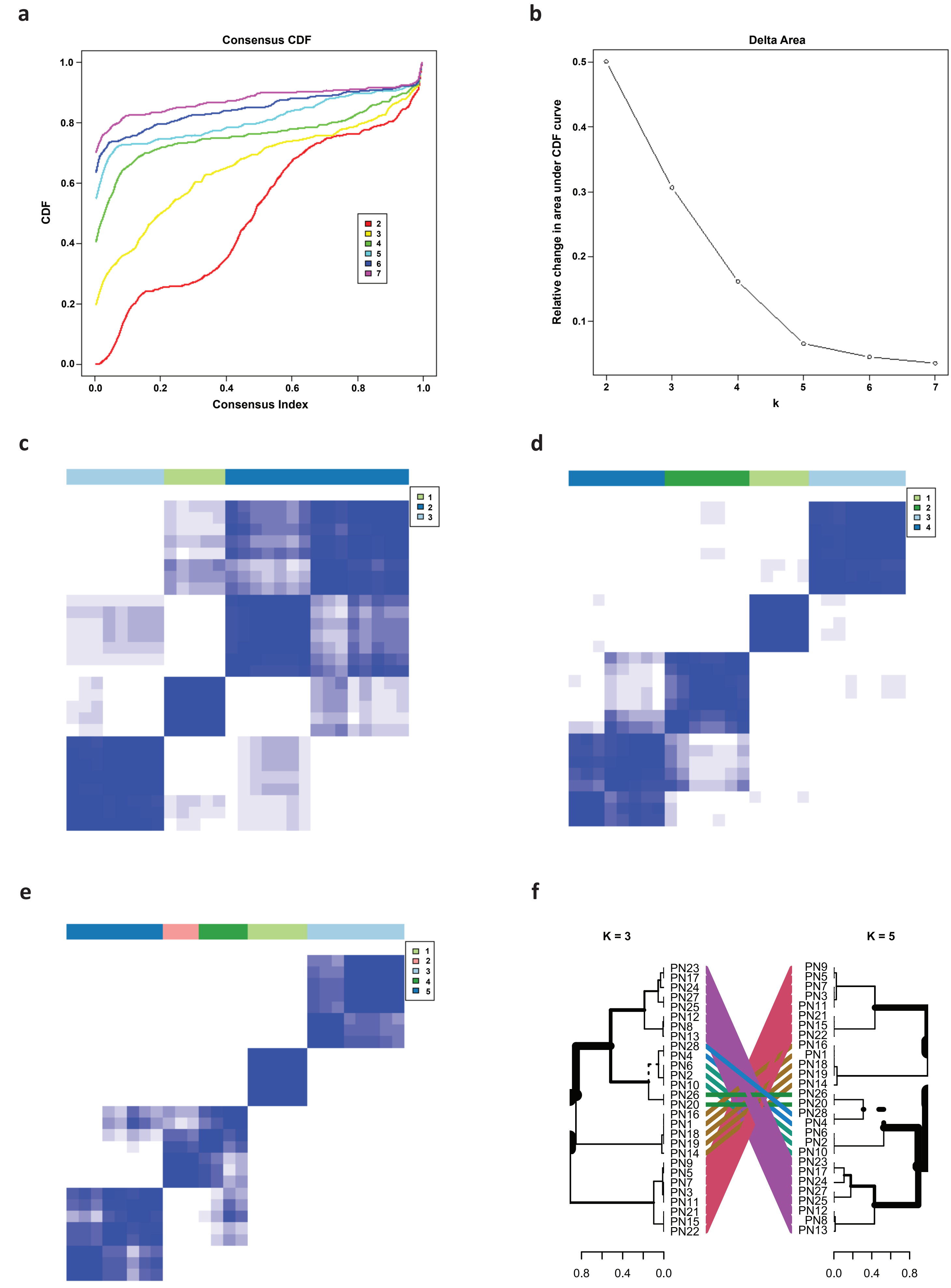

Supplementary Figure 3

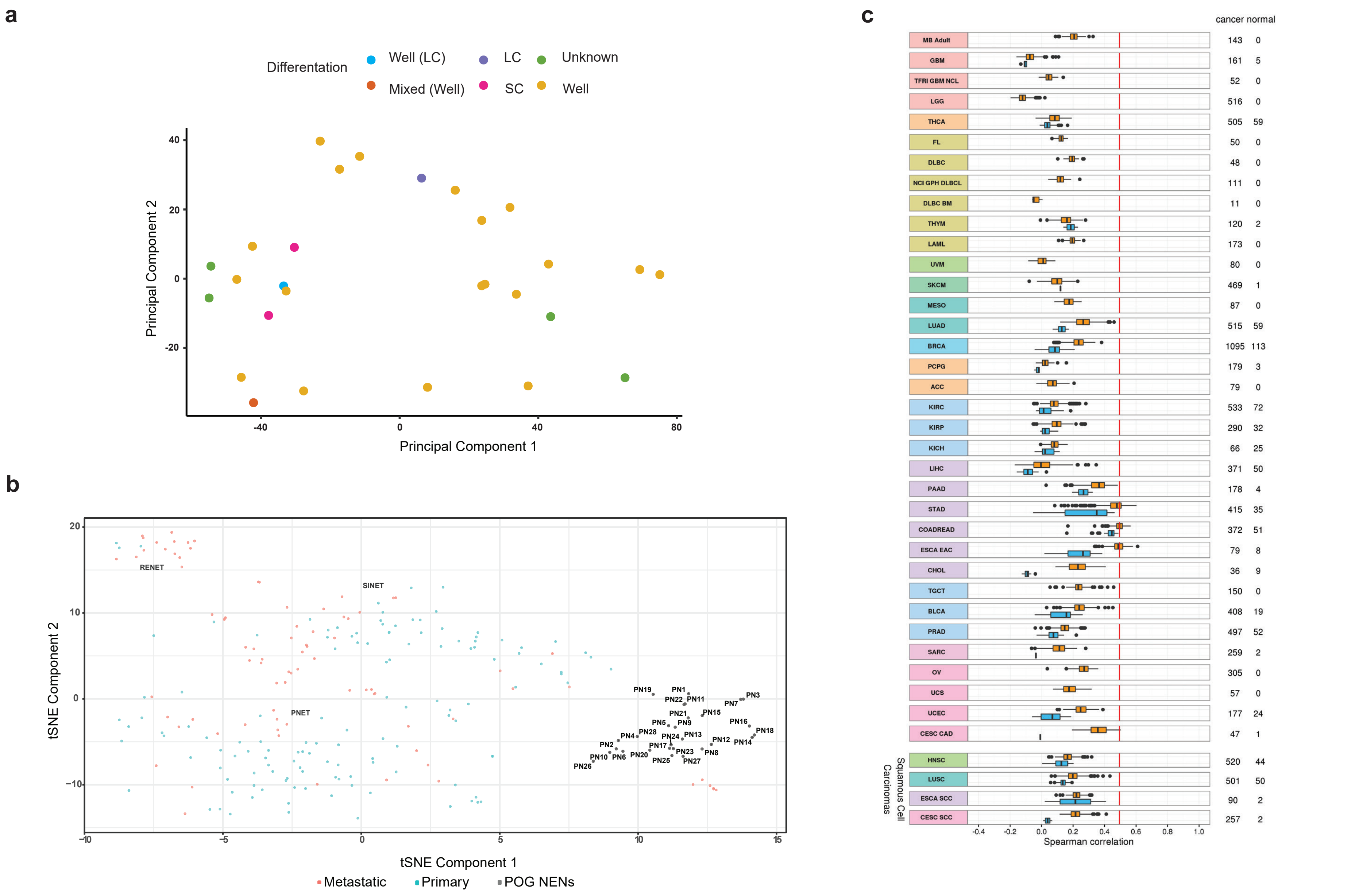

Supplement: Supplementary file 1 — Supplementary Information 1. [file 41598_2025_549_MOESM1_ESM.pdf]
